# Microfluidic Overhauser DNP chip for signal-enhanced compact NMR

**DOI:** 10.1038/s41598-021-83625-y

**Published:** 2021-02-25

**Authors:** Sebastian Z. Kiss, Neil MacKinnon, Jan G. Korvink

**Affiliations:** Institute of Microstructure Technology, Karlsruhe Institute of Technology, Hermann-von-Helmholtz-Platz 1, 76344 Eggenstein-Leopoldshafen, Germany

**Keywords:** Mechanical engineering, Electrical and electronic engineering, Microfluidics, Solution-state NMR

## Abstract

Nuclear magnetic resonance at low field strength is an insensitive spectroscopic technique, precluding portable applications with small sample volumes, such as needed for biomarker detection in body fluids. Here we report a compact double resonant chip stack system that implements in situ dynamic nuclear polarisation of a 130 nL sample volume, achieving signal enhancements of up to − 60 w.r.t. the thermal equilibrium level at a microwave power level of 0.5 W. This work overcomes instrumental barriers to the use of NMR detection for point-of-care applications.

## Introduction

Significant research effort has been invested in recent years to achieve nuclear magnetic resonance (NMR) systems possessing small form factors^1^. Compact NMR systems based on permanent magnets offer attractive opportunities, including a significant reduction in size and cost for hardware, and adaptable system operations, when compared to state-of-the-art high field NMR spectrometers based on superconducting electromagnets^2^. Only recently, technological progress has culminated in commercially available compact systems for NMR spectroscopy, based on permanent magnets, which provide sufficient performance for a variety of NMR and magnetic resonance imaging (MRI) applications. Micro NMR systems based on CMOS integrated circuits^3–6^ as well as advances in micro-fabrication and lab-on-a-chip technologies^7,8^ are further advancing miniaturisation, ultimately evolving towards handheld systems suitable for point-of-care and personalised diagnosis. Particularly, microfabricated NMR probes^9–17^ can provide the efficient analysis of volume limited samples and even co-integrate new functionalities to enable novel analytic approaches, such as NMR in situ electrochemistry.

Particularly the point-of-care diagnosis of certain devastating diseases, such as multiple drug resistant tuberculosis (TB), would benefit from both portability/miniaturization and the chemical specificity of NMR. A major barrier to progress has been the lack of a suitable NMR-detectable disease marker, which was recently overcome with the discovery of a TB metabolomic biomarker profile in urine^18^, but the importance of early disease detection implies a low concentration level of metabolites, which in turn requires a high detection sensitivity.

The benefits of portable NMR thus remain in opposition to many of the technological challenges associated with the system integration of, e.g., the sample container, the radio frequency (RF) coil, and the NMR transceiver. Importantly, the benefits of compact permanent magnets come at the price of low magnetic field strengths (less than 2 T for NdFeB magnets), resulting in low NMR signal sensitivity and poor $B_0$-field homogeneity, as well as thermally and mechanically induced $B_0$-field drift.

Overcoming these limitations, to enable high chemical shift resolution (< 0.01 ppm) NMR spectroscopy in a compact format, is challenging and the subject of ongoing research. Efforts to address the issues related to the magnet include: design optimisation of the magnet topology^19–24^, the implementation of passive shims^25^ and active electric shim coils^26–29^, and frequency/field locks for $B_0$-field correction and stabilisation.

With this contribution we specifically address the lack of low field sensitivity, by employing out-of-equilibrium polarisation enhancement of liquid state nuclear spins. Dynamic nuclear polarisation (DNP) is a strongly hyperpolarising effect that was first predicted by Albert Overhauser^30^ and soon afterwards confirmed by Charles Slichter and his team^31^, but, despite its advantages, is still not generally available. By miniaturising the DNP detection system, the opportunities offered by increased portability stand in contrast to numerous instrumental challenges that must be overcome. Inhomogeneities in low weight low field magnets result in significantly broadened NMR linewidths. Microwave (MW) and RF signals for DNP differ considerably in wavelength, making double resonance hard to achieve in an overlapping sample volume. Remanent MW electric field components lead to rapid sample heating due to water absorption. Microwave equipment with a miniaturised footprint delivers insufficient power output for typical sample volumes.

The existing literature focuses on conventional fabrication technologies and sample volumes of several microlitres to millilitres. For example, Armstrong and co-workers^32^, presented a portable X-band (9.5 GHz) system for solution-state Overhauser DNP (ODNP) based on a 0.35 T Halbach permanent magnet. A home-built NMR double U-coil was implemented into a modified commercial ${\mathrm {TE}}_{102}$ X-band MW cavity resonator, which was loaded by a capillary containing $4~{\upmu \hbox {L}}$ of sample. Münnemann et al.^33^, reported on a mobile DNP polariser based on a 0.3 T permanent magnet for clinical applications. The field-tunable Halbach magnet weighs 90 kg and is equipped with a commercial electron nuclear double resonance (ENDOR) probe head featuring a dielectric MW resonator, accepting 3 mm sample tubes. Garcia and co-workers^34^ presented an L-band (1.1 GHz) loop-gap MW resonator inside a solenoid NMR detection coil, for ODNP ${}^{1}{\mathrm{H}}$ experiments on water dynamics employing a 0.04 T permanent magnet of Halbach design and 8 mm NMR tubes. For a comparative study of ${}^{1}{\mathrm{H}}$ and ${}^{19}\hbox {F}$ ODNP in flourinated benzenes, Neudert et al.^35^, employed a nitrogen gas cooled, commercially available ENDOR probe head for operation at 9.69 GHz inside a 0.345 T home-built permanent magnet array^36^. The authors used sealed glass tubes, filled with approximately $13~{\upmu \hbox {L}}$ of sample. Keller et al.^37^ recently demonstrated chemically resolved ${}^{1}{\mathrm{H}}$ NMR ODNP operating at 0.35 T using a home-built dielectric resonator, accommodating a sample capillary with 1 mm diameter. This was possible by including field shimming coils, permitting 4 Hz spectral resolution. Überrück et al.^38^ recently described a benchtop ODNP system based on a home-built C-shaped permanent magnet at 0.342 T (15 kg) and commercial dielectric electron paramagnetic resonance (EPR) resonator, compatible with 1 mm sample capillaries. With this system, the authors also demonstrated ${}^{1}{\mathrm{H}}$ chemical shift resolution by shimming through adjustment of the unit permanent magnet positions.

Inspired by the work of Johansson et al.^39^ and Narkowicz and co-workers^40^, this work aims to explore the feasibility of microfabricated, 14 GHz transmission line MW resonators for ODNP experiments. We report on an NMR probe head enabling in situ Overhauser DNP-enhanced NMR spectroscopy of nanoliter-sized liquid samples for analyses inside a compact, light weight and hence portable 0.5 T permanent magnet. The approximately 3.5 mm long and $411~{\upmu }\hbox {m}$ wide $\lambda /2$-stripline resonator is integrated into a microfluidic chip and interacts with a sample volume of approximately 130 nL. Miniaturised, distributed resonators feature large power-to-field conversion efficiencies $\Lambda$ and high filling factors, beneficial for experiments on volume limited samples. The following section on the system design summarises the challenges and potentials involved with the design of the double resonant ODNP probe head. In particular, we also describe our MEMS-based approach to co-integrate a MW resonator into a microfluidic chip, including the implementation of the electrical shims and an RF transceive coil. We then demonstrate the performance of our probe head by performing $^{1}{\mathrm{H}}$ ODNP-enhanced NMR experiments and discuss benefits and disadvantages of the proposed concept. To the best of our knowledge, this is the first report on a compact microfabricated MW resonator integrated into a permanent magnet ODNP setup.

**Figure 1:**
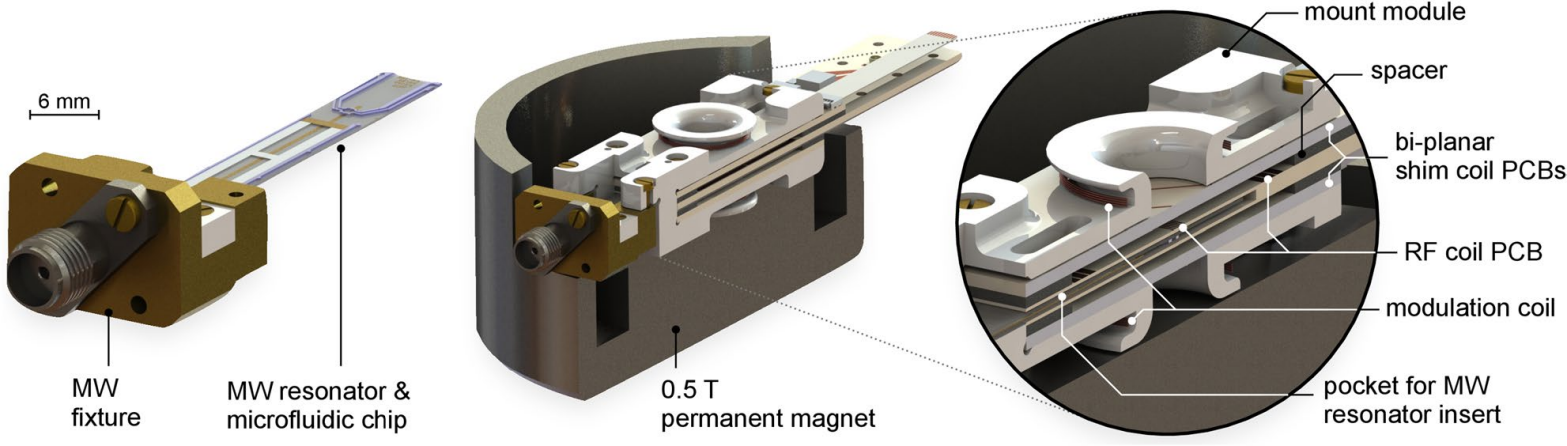
Computer aided design (CAD) views of the ODNP probe inside a palm-sized permanent magnet. The microfluidic chip features a sample reservoir and a MW resonator. A stacked figure-8 type RF transceive coil accepts the sample container and is sandwiched between a set of bi-planar electrical shim coils. A $B_0$-field modulation coil is part of phase sensitive EPR detection. Figure used with permission^41^.

### System design

Figure 1 illustrates all major components of the ODNP probe head. The probe was formed from a stacked assembly, which inserted into a 0.5 T parallel-plate, permanent magnet. A figure-8 shaped, open topology Tx/Rx coil was used for RF excitation and NMR signal acquisition. A set of miniaturised bi-planar electrical shim coils reduced the $B_0$-field inhomogeneities, as required for NMR spectroscopy. Nano-litre sized sample volumes were handled by a microfluidic chip that featured a co-integrated transmission line MW resonator to facilitate ODNP and EPR experiments. For phase sensitive EPR detection, a Helmholtz-type modulation coil was implemented. Our system design considerations addressed the following points:

- **Magnet effects**. The concept accounts for typical characteristics of permanent magnets, such as low thermal polarisation, typically poor $B_0$-field homogeneity, temperature induced $B_0$-field drift, and limited space between the magnetic pole pieces.
- **EM-field effects**. For an in situ ODNP system, the RF resonator, as well as the MW resonator, access the same sample volume, ideally without compromising performance. Therefore, the resonator topology allows sample excitation at both high (several GHz) and low (several MHz) frequencies, as needed for ODNP experiments. To minimise cross-talk, the MW-field direction is orthogonal to the direction of the RF-field, decoupling mutual inductive pathways.
- **Modularity**. The ODNP probe permits a high level of integration while still remaining modular. The concept handles a sub-microliter sample volume and is envisioned to be scalable in dimension, allowing to adapt to diverse applications. The targeted design ultimately avoids manual manufacturing, and thus is producible by automated fabrication methods.

## Results

### Resonator characterisation

For electrical characterisation, the resonator chips were mounted into the MW fixture as shown in Fig. 6b, which was connected to a calibrated network analyser (N5224A, Agilent Technologies, Inc., USA) via a 50$\Omega$ coaxial cable. Figure S3 shows characteristic features of the presented double resonant ODNP probe head, such as the frequency tuning capability, or the typical shift in MW resonance frequency $\Delta f_{\mathrm {L}} \approx -300$ MHz upon sample loading (panel b, top). The sample’s Larmor frequencies are determined in value by the $B_0$-field of the 0.5 T permanent magnet, which can only be marginally tuned via its built-in drift compensation coil, so that the ability to tune the MW resonator’s resonance frequency is of particular importance. MW frequency tuning is achieved by introducing a thin strip of polyimide, featuring a gold coated tip, between the top and bottom glass substrate of the MW resonators (maximal attainable tuning is $\Delta f_{\mathrm {e}} \approx-0.55$ GHz). In fact, the gold patch creates an additional capacitive pathway for the high frequency signals, effecting both frequency tuning, and impedance matching. This method is very sensitive to the positioning of the gold coated tip of the tuner. As the effect is of capacitive nature, it is most pronounced at locations of high electric field strengths, primarily above the coupling gap region of the MW resonator, less so along the microstrip transmission line. As apparent from Figure S3 (a), the change in frequency is unidirectional only, as the induced change in capacitance is positive (the capacitance is increased) the resonance frequency can only be lowered. The inset shown in the supplementary material in Figure S3 (a) illustrates the influence of the tuner’s position on the amplitude of the ${\mathrm {S}}_{11}$-parameter, indicating different degrees of impedance matching.

**Figure 2:**
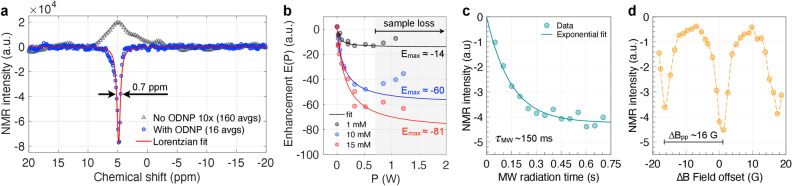
(**a**) Comparison between a thermal (160 averages, 10× scaled) and the ODNP enhanced ${}^{1}{\mathrm{H}}$ spectrum (130 nL of 15 mM TEMPOL in DI water, $P_{\mathrm {MW}}\approx50$ mW, 16 averages). (**b**) Measured ${}^{1}{\mathrm{H}}$ ODNP enhancements as a function of the applied MW input power. Three concentrations of TEMPOL in DI water are shown (sample volume always 130 nL). (**c**) ODNP build-up curve as a function of MW radiation time (see text for details). (**d**) Measured ${}^{1}{\mathrm{H}}$ ODNP enhancement profile. Shown are the NMR signal peak values plotted versus the available $\Delta B$-field range of the $B_0$-field sweep coil (centre $B_0$-field strength was 4940 G).

The MW resonator as well as the RF resonator access the same sample volume. As a consequence, two obvious but important requirements need to be fulfilled: (i) the electromagnetic (EM) performance of the MW resonator should not be compromised, once inserted into the RF coil, and closely related (ii) the RF performance of the NMR Tx/Rx coil should not be impaired by the presence of the MW resonator either. For (i), the ${\mathrm {S}}_{11}$ measurements shown in Figure S3 (b) (bottom panel) prove that the MW resonance of the MW resonator is not spoiled once inserted into the RF coil assembly. This is achieved by two design choices, firstly, both resonators are geometrically decoupled ($B_1$ fields orient orthogonal to each other), and secondly, the aspect ratio of the conductors is very low, limiting the formation of eddy currents substantially. Results on point (ii) are given in the subsection further below. As described below, apart from the MW resonator’s length parameter *L*, the geometry parameters constituting the coupling gap region are crucial and effect both the resonance frequency as well as the impedance matching. Figure S4 (a) shows the influence of the finger length $C_{\mathrm {l}}$ on the resonance frequency $f_i$ for a fixed, nominal coupling gap width of $C_{\mathrm {gap}} =25~{\upmu }\hbox {m}$. Resonance frequencies and the loaded Q-factors $Q_{\mathrm {L}} = f_0 / (f_2 - f_1)$ were determined from the ${\mathrm {S}}_{11}$ curves measured by a network analyser. Measured values are compared to driven numerical EM simulations, with model properties as given in Table S1. Average measured (simulated) Q-factors are 33 (23) for the type 2 resonator. Assuming critical coupling ($\kappa = 1$), the unloaded measured Q-factors $Q_0 = Q_{\mathrm {L}} (1 + \kappa )$ can be determined to be 66, which agrees reasonably well with the value of 75 as estimated (see supplementary material Section 1). However, most of the investigated MW resonators are under-coupled ($\kappa < 1$), suggesting the presence of unaccounted loss mechanisms due to, e.g., surface roughness, radiation loss, and unconsidered high frequency dielectric losses of the involved materials.

### ODNP-enhanced ${}^{1}{\mathrm{H}}$ NMR

**Figure 3:**
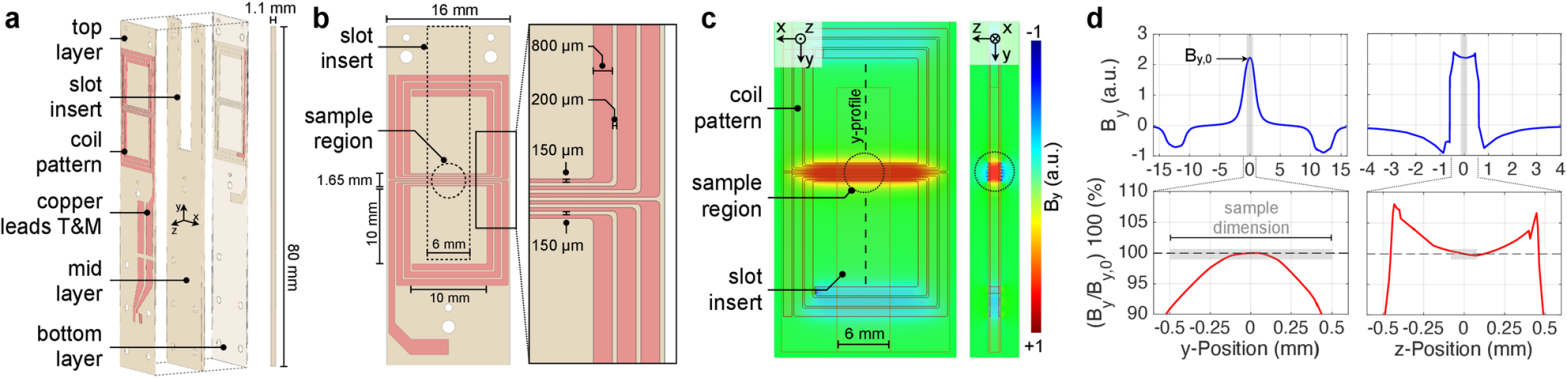
RF coil geometry and 3D EM field simulations at self-resonance (ca. 48 MHz). (**a**) Exploded view model of the stacked figure-8 coil. The total height of the pressed PCB stack is approximately 1.1 mm. A $6~\hbox{mm}\times30~\hbox{mm}$ sized slot is milled into the mid layer spacer (ca. 0.8 mm in height) to accept the fluidic insert featuring the MW resonator. (**b**) Zoomed-in details of the coil traces. The slot and sample region are indicated by dashed lines. (**c**) Density plot (top- and cross-sectional view) of the $B_{\mathrm {y}}$-field component, showing three distinct high magnetic field regions due to in-phase field superposition. (**d**) $B_{\mathrm {y}}$-field profiles along the y- ($x=z=0$) and z-direction ($x=y=0$) of the coil, as well as normalised data shown over the sample region (gray rectangles). Figure used with permission^41^.

The development of high performance double resonant structures, for in situ, resonant DNP probe heads, presents a central technical challenge. In the above subsection, one-port ${\mathrm {S}}$-parameter measurements confirm (i), i.e., that the EM performance of the MW resonator is not spoiled upon inserting the chip into the dedicated slot inside of the RF coil (see Fig. 3). The above mentioned point (ii) is addressed by data shown in Figure S4 (b). The figure compares the ${}^{1}{\mathrm{H}}$ NMR spectra obtained by using a bare glass chip (no metallisation) with the spectrum acquired by using an actual metallised MW resonator chip. Except from a spectral shift, both spectra are identical, confirming that the excitation/detection performance of the NMR coil is not impaired due to an inserted MW resonator chip. Following the measurement procedure given in the supplementary materials, a set of basic ${}^{1}{\mathrm{H}}$ ODNP experiments was conducted to determine polarisation enhancements. The ODNP enhancement *E* is given by the ratio $E = \langle {\hat{I}}_{\mathrm {z}} \rangle / I_0$, with $\langle {\hat{I}}_{\mathrm {z}} \rangle$ being the enhanced ${}^{1}{\mathrm{H}}$ signal and $I_0$ being the thermal equilibrium value. Figure 2a compares the thermal (MW off) ${}^{1}{\mathrm{H}}$ NMR signal to an ODNP-enhanced spectrum (MW on), acquired from 15 mM TEMPOL dissolved in 130 nL of DI water. At this concentration, the ODNP experiment shows a signal enhancement of $E\approx -40$, employing a MW input power level of $P_{\mathrm {MW}}\approx50~\hbox{mW}$ and carefully adjusted shims (type 2 MW resonator, $L=$ 3.635 mm, $C_{\mathrm {l}}=200~{\upmu }\hbox {m}$, $C_{\mathrm {gap}}=20~{\upmu }\hbox {m}$, $w_{\mathrm {c}}=100~{\upmu }\hbox {m}$). The signal enhancement was also computed from the measurements using Topspin 3.6 (Bruker, Rheinstetten). These values delivered the signal-to-noise ratio (SNR) term in the nLOD equation (eq. 5 in Badilita et al.^42^), yielding ${\text {nLOD}}_{\mathrm {mol(therm)}} = 120~{\upmu \hbox {mol s}^{1/2}}$, and ${\text {nLOD}}_{\mathrm {mol(ODNP)}}= 2.5~{\upmu \hbox {mol s}^{1/2}}$, which yields a computed enhancement of 48.

The NMR transmitter frequency was set to 21 MHz, using an RF input power of 2 W. A well shimmed ODNP-enhanced ${}^{1}{\mathrm{H}}$ spectrum features 0.7 ppm or 14 Hz full width at half maximum (FWHM) line width spectral resolution. The radical concentrations employed (up to 15 mM) did not lead to dominant broadening of the proton lines (also see Figure S5). Although the extraction of relaxation data could be challenging, Han and Franck et al.^43^ have outlined procedures to correct for DNP induced temperature changes as well as solvent/radical effects, and how to obtain quantitative data from ODNP experiments, so that this is not considered a principal obstruction. Noticeably, measured thermal spectra did not assume a clear Lorentzian-type line shape, whereas the ODNP-enhanced spectrum did. We attribute this discrepancy mainly to parasitic ${}^{1}{\mathrm{H}}$ NMR signal contributions originating from the microfluidic in-/outlets next to the sample reservoir, regions that are not sufficiently covered by the electrical shim system. The relative error for the determined thermal signal intensities was therefore estimated to be 10%, which translates into uncertainties for the enhancements $\Delta E$ of up to $\pm 6$. In order to reduce undesired sample heating effects due to CW MW irradiation, the start-stop timing of the MW source was controlled by TTL trigger signals of the NMR console. Figure 2c shows a polarisation build-up curve, representing the (negative) signal peak values determined from a series of ODNP spectra, acquired by successively increasing MW irradiation times $t_{\mathrm {rad}}$. The experiments were recorded using the same type 2 MW resonator at a fixed MW power of $P_{\mathrm {MW}}=130~\hbox{mW}$ from a 15 mM TEMPOL DI water sample. Fitting the signal growth (negative intensities) by employing the exponential model $a \left[ 1 - \exp (t_{\mathrm {rad}} / \tau _{\mathrm {MW}})\right]$, reveals a characteristic signal build-up time of $\tau _{\mathrm {MW}}=150~\hbox{ms}$. In order to establish steady-state conditions during each ODNP experiment, $t_{\mathrm {rad}}$ was typically kept in the range of 200–800 ms.

Figure 2b shows the ${}^{1}{\mathrm{H}}$ signal enhancements $E(P_{\mathrm {MW}})$ as a function of the applied MW input power, measured for three radical concentrations. The NMR acquisitions were performed at a transmitter frequency of 21 MHz, a repetition delay of 3 s, an RF power of 2 W, and a pulse length of $7~{\upmu }\hbox {s}$. The applied MW power levels, as set in decibel units at the MW source, were corrected for the employed attenuators and converted into units of Watt. As apparent from the diagram (gray region), the enhancements *E* deviate significantly from the expected fitting model, given by $1 - E = {a_1 P_{\mathrm {mw}}}/{(1 + a_2 P_{\mathrm {mw}})}$, for higher power levels. This effect is due to the loss of sample, caused by dielectric heating for power levels exceeding 0.5 W. To fit the data, only enhancements below 0.5 W were included. Figure 2d shows an ODNP enhancement profile, whose envelope resembles the EPR transitions of the ${}^{14}\hbox {N}$ radical. For this experiment the $B_0$-field was systematically varied using the built-in auxiliary coil of the magnet, while ensuring the correct tuning and matching of the RF coil.

## Discussion and Conclusion

Due to the low polarisation field of 0.5 T, bare-bones 21 MHz NMR struggled to deliver a reasonable ${}^{1}{\mathrm{H}}$ signal from the 130 nL small sample solution despite careful shimming, but Overhauser enhancement by microwaves at 14 GHz ($P_{\mathrm {MW}} =$ 0.5 W) could boost the SNR to up to − 60 in around $\tau _{\mathrm {MW}}=$ 0.15 s to 0.5 s of irradiation. This enhancement level observed for the present system is in reasonable agreement with work previously published by Armstrong et al.^32,44^, who obtained measured ${}^{1}{\mathrm{H}}$ enhancements of around − 50 to − 70 from sample solutions containing 15 mM and ${{}^{14}\hbox {N}}$ 4-amino-TEMPO and ${{}^{15}\hbox {N}}$ 4-oxo-TEMPO employing similar MW power levels but a 0.35 T electromagnet. For nLOD comparison of our work with similar reports, the limit of detection is often referred to 600 MHz, yielding ${\text {nLOD}}_{\mathrm {mol(ODNP/600)}}=$ 8.5 $\hbox {nmol s}^{1/2}$, which is an improvement on the value reported e.g. by Überrück et al.^38^ of approximately ${\text {nLOD}}_{\mathrm {mol(ODNP/600)}}=$ 150$~\hbox {nmol s}^{1/2}$(acetic acid, methyl group). To be fair, different molecular groups in a substrate will show differing enhancement values.

The EPR frequency at 0.5 T was very close to the absorption line of water, so that extended power irradiation eventually lead to heating of the sample, and evaporation, despite careful design of the resonator. This can be countered by operating the measurement at a less absorptive EPR/DNP frequency, or by active cooling of the sample during measurement, for example based on the Peltier effect.

The paper does not address the miniaturisation of the spectrometer, nor does it consider a compact solution for magnet stabilisation. Recent work by Jouda^45^ has explored EPR detection utilising a commercial GHz source chip by Analog Devices (e.g. ADF5610), and Anders et al.^46^ have demonstrated a CMOS chip oscillator reaching 14 GHz that yields sufficient power for the EPR excitation of a $200~{\upmu }\hbox {l}$ sample, and enough sensitivity for detection. Very recently the Boero group^47^ presented a CMOS based single-chip DNP system for experiments inside an electromagnet. The authors conclude that employing an all planar topology is limited in terms of NMR excitation homogeneity and confirm that the pronounced EM coupling between the MW and RF resonant structures in their setup represents a major technical challenge. By suitable insulation and power management based on available technologies, the feasibility of magnet thermal stabilisation is also not considered to be technically demanding.

The compact probe head of this report was based on wafer scale microfluidic chip processing, precision multilayer PCB manufacturing for the shim coils, combined with fused-deposition 3D printing for the holder and sweep coils, and was designed to fit snuggly into the commercial 0.5 T palm-top NMR magnet. The tight integration was especially facilitated by the high degree of $B_1$-field orthogonality between the MW and RF resonators, thus suppressing coupling, nevertheless did not compromise the system’s scalability w.r.t. sample volume, or its modularity of seperate functional components, such as shims, sweep coils, resonators, and microfluidics.

The system’s chemical shift resolution of 0.7 ppm compared favourably to the recent reports by Keller et al.^37^ (0.3 ppm using a hybrid magnet after active shimming) and Überrück et al.^38^ (3.6 ppm ${}^{1}{\mathrm{H}}$ NMR spectrum of acetic acid with passive shims), whilst maintaining a high level of system compactness, and a low magnet weight.

This work thus clearly shows, for the first time, the feasibility of Overhauser DNP for a mass-producable microfluidic assay in an ultra compact arrangement.

## Materials and Methods

### RF resonator

For NMR excitation and detection, a stacked layer coil topology was targeted. As sketched in Fig. 3, the micro-inductor consisted of two vertically aligned coil traces of six turns each, resulting in an open volume coil geometry with a total number of 12 turns. The top and bottom coil patterns resemble the shape of two figure-eights, separated by an $$800~
{\upmu }\hbox {m}$$ thick spacer. Variable conductor trace widths reduced excessive eddy current losses^48,49^ and improved the quality factor of micro 2D inductors, here achieved by employing two track widths. The outer metal traces were chosen to be $$800~{\upmu }\hbox {m}$$; for the centre region the trace width was uniformly reduced by a factor of $$5.3~\hbox{to}~150~{\upmu }\hbox {m}$$. This served two purposes, (i) it concentrated all traces aligned with the y-axis within a ± 0.825 mm zone, overlapping the lateral dimension of the sample reservoir, and (ii) it increased the current density and hence $$B_{\mathrm {y}}$$-field magnitude in that region.

As apparent from Fig. 3b, a $6~\hbox{mm}\times 30~\hbox{mm}$ slot was left open in the mid (spacer) layer of the stack in order to accommodate a microfluidic chip, featuring the MW resonator and sample reservoir. The RF excitation region was localised at the geometric centre of the figure-8 shaped coil pattern between the top and bottom planes. In this region, parallel coil traces guide currents in opposing direction on each face of the sample, resulting in a constructive $B_1$-field superposition. Compared to a single layer coil design, the stacked approach offers several advantages, including a higher overall inductance, and improved $B_1$-field homogeneity and magnitude, and low stray field components. The open coil topology accommodated the microfluidic chip, in a way that both RF and MW resonator were electromagnetically decoupled by their orthogonal geometric arrangement. The resonant coil’s $B_1$-field homogeneity at 48 MHz, as well as its electrical characteristics, were confirmed by finite-element EM field simulations, shown in Fig. 3c,d.

The CAD model of the coil was imported into the simulation software (HFSS, version 16, ANSYS) as a sheet conductor implementing a finite conductivity boundary condition (copper). The coil was modelled inside a box-shaped air volume with an outer radiation absorption boundary. The structure was excited using a lumped port with a reference impedance of 50$\Omega$. The $B_{\mathrm {y}}$-field density plot (Fig. 3c) reveals three distinct enhanced-field regions, caused by the in-phase field superposition at coil traces aligned along the x-direction, and a more than tripled $B_{\mathrm {y}}$-field magnitude at the position of the sample reservoir. Figure 3d also reveals the excellent $B_{\mathrm {y}}$-field homogeneity $B_{\mathrm {y}} / B_{\mathrm {y,0}}$, with $B_{\mathrm {y,0}} = B_{\mathrm {y}} (x, y, z = 0)$. The geometry of the employed sample container is approximately a thin disc of low aspect ratio $\eta = d_z/d_x = 0.2$ (illustrated by gray regions within the diagrams). Therefore, inhomogeneities along the z-direction contribute a factor $\eta$ less to the NMR spectral line broadening than those present along the x,y-directions.

### MW resonator and microfluidic insert

**Figure 4:**
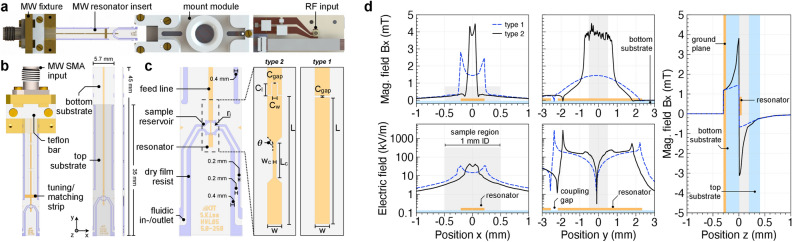
(**a**) Topview of the ODNP probe head model, shown with the MW resonator detached. (**b**) Overview of the MW fixture and the microfluidic chip. (**c**) Geometric details of the two designs of microstrip resonators (ground-plane not shown). (**d**) Electric and magnetic field profiles as extracted from the EM simulations for resonator type 1 and 2. Profiles are shown along the corresponding axes. As a guide to the eye, the lateral extent of the metallisation of the resonator (depicted in orange) and sample (grey) are indicated in the diagrams. Figure used with permission^41^.

For ODNP experiments, the electron spin transitions of radicals inside the sample are excited and ideally driven into saturation by means of MW radiation. Figure 4a illustrates the ODNP probe head design and its microstrip line MW resonator integrated into a $45\times5.7\times0.675~\hbox{mm}^{3}$ $({\mathrm {l}} \times {\mathrm {w}} \times {\mathrm {h}})$ microfluidic chip. In the figure, the MW resonator is shown detached from the RF coil. When inserted into the slot of the RF coil, the fixture is attached and held in place by the 3D-printed mounting module. The rectangular glass chips depicted in Fig. 4b were mechanically fixed and electrically connected onto a brass fixture. A Teflon bar provided electrical compression contact between the inner conductor of the SMA connector and the MW launch point of the transmission line. Figure 4c shows geometrical details of the fluidic channels, as well as the transmission line MW resonator designs considered. Important layout parameters, such as the resonator width *w*, length *L*, as well as the coupling geometry, were analytically determined and served as a reasonable starting point for further EM simulations.

Two microstrip designs were investigated. Type 1 is a standard $\lambda /2$-resonator. Type 2 is also a $\lambda /2$-resonator, but featuring a symmetric constriction. To maintain alignment of the centre of the MW resonator to the centre of the $B_1$-field of the RF coil, as well as the sample volume for $B_0$-field shimming, the distance between the MW launch point of the microstrip feed line and the MW resonator’s centre point (*L*/2) was kept fixed at 35 mm for all design variations. As a consequence, the length of the feed line varied for changing resonator lengths. The resonator was operated as a half-wavelength, open-circuited resonant strip of length *L*. The resonance frequency $f_0$ of the fundamental mode was primarily adjusted by the choice of *L* which, neglecting fringing effects, can be estimated by

1 $$\begin{aligned} L = m \frac{\lambda }{2} = m \frac{c}{2 f_0 \sqrt{\epsilon _\ell }}, \end{aligned}$$

with the guided wavelength $\lambda$, speed of light *c*, integer mode number $m = 1,2,3,\ldots$, and effective dielectric permittivity $\epsilon _\ell$. For a targeted resonance frequency of 14 GHz ($m = 1$) and $\epsilon _\ell = 4.52$, the required length of an uncoupled resonator is approximately 5 mm. The width *w* of the resonator was chosen to be equal to the width of the feed line, whose characteristic impedance $Z_0 = 50$ $\Omega$ was determined by known equations^48^. For the employed borosilicate glass substrate of $h =0.33~\hbox{mm}$ in height, a characteristic impedance of 50$\Omega$ is met for the ratio of $w/h =1.47$, which resulted in a track width of $w = 0.441~\hbox{mm}$. Table S2 provides an overview of the involved geometry and material parameters. The supplementary materials present the detailed resonator design following standard electrical engineering procedures, as well as the characterisation using field simulations.

For electron paramagnetic resonance, an ideal-performing resonator topology should maximise the MW conversion efficiency $\Lambda = B_{\mathrm {MW}} / P^{1/2}$ that penetrates the sample, and should ensure EM field separation with low sample penetration of the electric field, to minimise dielectric sample heating. Polar and electrically conductive sample solutions, in particular, absorb MW power $P_{\mathrm {abs}}$, which scales as $P_{\mathrm {abs}} \propto \epsilon |E_{\mathrm {MW}} |^2$, with $\epsilon$ being the sample permittivity, and can cause significant increases in temperature and sample evaporation. The field profiles shown in Fig. 4d illustrate this characteristic. High E-field magnitudes are present at the two opposite sites of the resonator, particularly across the capacitive coupling region. At resonance, the magnetic $H_{\mathrm {x}}$-field maximum is found to be at the sample location, i.e., the resonator’s centre.

In comparing the field profiles for both resonator types, Fig. 4d reveals significantly higher magnetic field peak values $H_{\mathrm {xy,max}}$ for resonator type 2 (1.6 and 3.1 times higher than that for type 1 along the x and y axes). As expected, the geometric constriction of type 2 lead to higher current densities and an increased magnetic field, covering a stripe across the 1 mm sample diameter. However, in absolute terms this came at the price of considerably higher E-field strengths (1.3 times higher along the x-axis, 2.7 times higher along the y-axis) penetrating the cylindrical sample region. From the EM simulation, the power to field conversion factor can be estimated to be $\Lambda _{\mathrm {type~1}} \approx {2}\,\hbox {mT} \hbox {W}^{-1/2}$ and $\Lambda _{\mathrm {type~2}} \approx {4.5}\,\hbox {mT} \hbox {W}^{-1/2}$. To quantify the performance indicators, the field energy ratio $\Gamma$ around the sample region was determined from the profiles presented in Fig. 4d, given as

2 $$\begin{aligned} \Gamma = \frac{\mu _0 }{\epsilon _0}\frac{\int H^2_{\mathrm {x}} ds}{ \int E^2 ds}. \end{aligned}$$

Interestingly, $\Gamma$ for both resonators along the x- and y-axes are found to be remarkably similar (approximately $\Gamma _{\mathrm {x}}=2.6\times10^{14}$ and $\Gamma _{\mathrm {y}}=1.0\times10^{14}$). The integration interval of the line integrals was placed symmetrically across the geometric centre of each resonator, with an upper and lower limit of plus/minus the sample diameter (± 0.5 mm). These results suggest that type 2 resonators yield superior $H_{\mathrm {x,max}}$ values, while offering similar performance to frustrate the electric field around their centre region, when compared to type 1 resonators. In other words, to achieve similar $H_{\mathrm {x,max}}$ values, a type 1 resonator would require higher MW input power to match the vales obtained by type 2. Alternatively, a reduced *L* of type 1 resonators might compensate for that disadvantage. However, this also would push the E-field maximum closer towards the sample region than is the case for the type 2 topology.

### Permanent magnet

**Figure 5:**
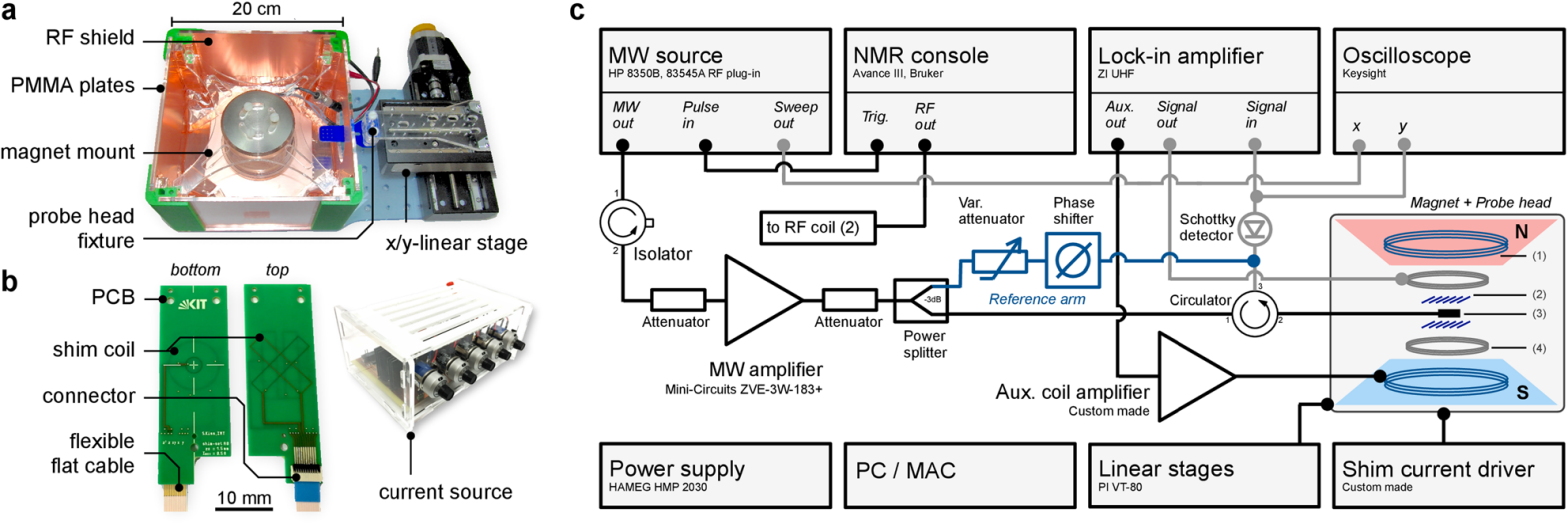
(**a**) The 0.5 T permanent magnet is suspended inside an RF-shielded box for environmental isolation. For precise positioning at the magnet’s most uniform spot, the probe was mounted on a motorised x/y linear stage. (**b**) Top and bottom of the fabricated PCB-based, five channel, bi-planar shim coils as well as the custom made shim current driver. (**c**) Schematic of the ODNP measurement setup. The reference arm (blue) as well as the one-port reflectometry setup (gray) are optional and were not permanently connected. The magnet + probe head panel includes, (1) $B_0$-field coil of permanent magnet, (2) RF coil, (3) MW resonator and (4) EPR modulation coil. Figure used with permission^41^.

For ODNP experiments a palm-sized, parallel-plate type, 0.5 T NMR permanent magnet (PM-1055-050N, Metrolab Instruments SA, Switzerland) was used. The cylindrically shaped magnet had an outer diameter of 8 cm and featured an air gap between two NdFeB-poles, separated by approximately 11 mm, and thus defining the maximum height of the probe head assembly. $B_{\mathrm {0}}$-field adjustments, as well as field-sweeps, were accomplished via a built-in auxiliary coil, which provided an adjustment range of $\Delta B_{\mathrm {aux}}= {\pm 3.75}\,\hbox {mT}$, which translates into $\Delta f_{\mathrm {e}}\approx {\pm 98}\,{\hbox {MHz}}$ and $\Delta f_{\mathrm {^1H}}\approx {{\pm 150}\,{\hbox {kHz}}}$ in terms of EPR and ${}^{1}{\mathrm{H}}$ NMR frequencies, respectively. As shown in Fig. 5a, the magnet was mounted inside a Faraday cage box, providing RF-shielding, as well as a thermally stable environment. For optimal NMR performance, the location of a region inside the magnet with minimal magnetic field gradient was determined from an accurate $B_{\mathrm {0}}$-field map using a Hall probe (DTM-151, Group 6, New Zealand) and an x/y-linear stage (VT-80, Physik Instrumente GmbH, Germany). To improve measurement reproducibility and to reduce thermally induced $B_{\mathrm {0}}$-field drift (${-\,1200}\,\hbox {ppm}\, \hbox {K}^{-1}$ or a shift of about ${-\,24}\,\hbox {kHz}\, \hbox {K}^{-1}$ in $^1\hbox{H}$ Larmor frequency), a temperature-controlled fan heater was employed, raising the magnet’s temperature stably above room temperature (see Figure s1).

### Bi-planar shim coils

The NMR line broadening due to permanent magnet field inhomogeneity of several kHz are inacceptable when compared to spectrograms obtained from shimmed high field magnets (sub 1 Hz). NMR systems based on permanent magnets commonly require multiple electrical shim coils in order to restore homogeneity. In this work, a five channel bi-planar shim coil set was implemented that is based on a design originally proposed by Anderson^50^ (implemented shim orders: ${\mathrm {x}}$, ${\mathrm {y}}$, ${\mathrm {z}}$, ${\mathrm {z^2}}$, $\mathrm {xy}$), placed between the magnet poles to correct for $B_{\mathrm {0}}$-field distortions. The shims were positioned 3 mm apart in the ${\mathrm {z}}$-direction and covered a spherical shim region with a radius of approximately 1.5 mm. Figure 5b shows the discrete shim coil track of a micro-fabricated shim PCB. Shims related to the ${\mathrm {x}}$ and ${\mathrm {y}}$ axes were implemented as rectangular coils, whereas the ${\mathrm {z}}$ and ${\mathrm {z^2}}$ shims used circular traces. Care was taken to adjust the geometry of each shim to account for its ${\mathrm {z}}$-position within the multilayer PCB. Whenever possible, current return paths were routed to line up with the input current paths, in order to reduce undesired magnetic stray fields and noise-pickup effects.

### ODNP setup and signal processing

A block diagram of the EPR and NMR detection system is shown in Fig. 5c. The developed probe head was mounted on an x/y-linear stage (VT-80, Physik Instrumente GmbH, Germany) controlled by a PC and its position precisely maintained inside the magnet. The auxiliary signal output (${{\pm 10}\,{\hbox {V}}}$ at 50 $\Omega$, $I_{\mathrm {max}}=100~\hbox{mA}$) of a lock-in amplifier (UHFLI, Zurich Instruments, Switzerland) connected to the input of a custom-built amplifier, which drove the magnet’s auxiliary coil. The remainder of the measurement setup/procedure is standard and described in the supplementary materials.

### Sample preparation and loading

**Figure 6:**
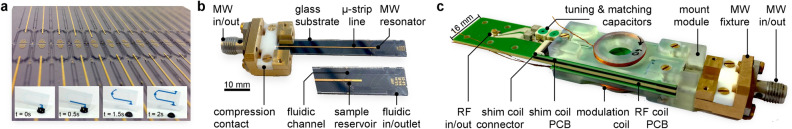
(**a**) Photograph of a bonded wafer stack featuring 30 individual microstrip resonators. The insets demonstrate the loading of aqueous sample (colored by blue pigments) via capillary forces. (**b**) Fabricated MW fixture, equipped with a resonator chip. (**c**) Fabricated ODNP probe head, shown with the MW microfluidic chip being inserted into the stacked figure-8 NMR transceive coil. Figure used with permission^41^.

A 100 mM 4-hydroxy-TEMPO (2,2,6,6-tetramethylpiperidyl-1-oxy, Sigma-Aldrich, $\hbox {C}_9\hbox {H}_{18}\hbox {NO}_2$, CAS number 2226-96-2, molecular weight $171.22~\hbox {g mol}^{-1}$) stock solution, dissolved in non-degassed purified water was prepared and stored in a $-20^{\circ }$C laboratory fridge. Working aliquots with concentrations of 1, 10 and 15 mM each were freshly prepared for measurements from the stock solution. For sample loading, a small amount of radical solution was pipetted onto a clean hydrophobic surface (Parafilm 3M, laboratory film) forming an hemispherical droplet, which was carefully brought into contact with the inlet of the fluidic microchip. As shown in the inset of Fig. 6a, the hydrophilic fluidic channels were rapidly filled by the aqueous sample solution via capillary force, without trapping any air bubbles. Reversible sealing of the in- and outlet of the chip was difficult and was simply omitted for most of the measurements. However, best sealing results were achieved by employing a droplet of wax. Due to the change in the dielectric environment of the MW resonator upon sample loading, the resonator’s resonance frequency is influenced. The change in frequency $\Delta f_{\mathrm {L}}$ depended on the sample properties and, for an aqueous sample solution, could be a shift down to − 300 MHz. For each resonator, the resonance frequency was fine-tuned (see tuning/matching strip) as close as possible to the electron frequency-equivalent of the magnet’s static $B_{\mathrm {0}}$-field value (typically around 13.84 GHz).

## Microfabrication

### RF and shim coils

The stacked figure-8 NMR coil was realised as a high resolution PCB (PCB outline 80 mm by 16 mm, total thickness of ca. 1.1 mm), fabricated from high frequency, ultra-low loss laminates (R5775 Megtron 6, $\tan \delta = 0.004$ at 12 GHz, $\epsilon _{\mathrm {r}}= 3.6$, $T_{\mathrm {g}}\approx 185^\circ$C) in a 4 layer process. The tin coated copper tracks were $35~{\upmu }\hbox {m}$ thick with a minimal feature size of $100~{\upmu }\hbox {m}$. In order to accommodate the MW resonator chip, a similar-sized rectangular shape was cut out from the $800~{\upmu }\hbox {m}$ core layer. For the RF connection, a low profile surface mount 50 $\Omega$ micro coaxial connector (Molex, MCRF Series) was soldered to the PCB.

The five channel electrical shim set was realised as an 8-layer PCB, arranged in a bi-planar fashion. The multilayer stack comprised eight signal layers, each featuring $35~{\upmu }\hbox {m}$ thick copper tracks, chemically coated by tin. The vertical space in the z-direction of the magnet was limited, necessitating the use of a compact 10 pin surface mount connector (Molex, Easy-On, FD19 Series) accepting a flat flexible cable to connect the coils to the current sources. In order to ensure the correct separation in the z-direction, the shim PCBs were not directly attached to the RF coil PCB, but were suspended on each side (top and bottom) by a pair of spacers of defined height.

### Microwave fixture

The MW fixture, as shown in Fig. 6b was precision milled from non-magnetic brass. A 50 $\Omega$ stainless steel RF connector (Part Nr. 1052902-1, TE Connectivity) was mounted on the front panel of the fixture. To avoid extensive MW signal loss and reflection effects, the microstrip signal launch region was designed so that (i) the ground return path was continuous and minimal and (ii) impedance mismatches were minimised^51^. Addressing (i), the brass surface in contact with the microstrip ground plane was continuous and smooth, minimising surface resistance. For point (ii), the 50 $\Omega$ condition was maintained at the transitions between the connector and the fixture, as well as the connectors’ signal pin and the microstrip. For solderless and flexible mounting of the resonators, a low-loss dielectric bar made from Teflon was used to provide an electrically reliable compression contact.

### Microwave resonator

The microstrip line MW resonators were batch fabricated and defined by UV lithography (EVG 620, Mask Alignment System, Austria). Square shaped $101\times 101~\hbox {mm}^{2}$ borosilicate glass substrates (D 263 T eco, SCHOTT, $\tan \delta = 0.01$, $\epsilon _{\mathrm {r}} = 6.3$) of 0.3 mm thickness were cleaned and coated on their top and bottom faces with a chromium and gold layer (10 nm Cr, 50 nm Au) by physical vapour deposition. The gold layer provided an electrically conductive seed layer for the following electroplating step. Dicing tape (Ultron systems, Minitron GmbH) was laminated to the substrate’s bottom face for protection. A UV sensitive $10~{\upmu }\hbox {m}$ thick photoresist (SU-8 3005, MicroChem Corp.) was spin-coated and structured by UV light. Unexposed areas of the resist formed open windows after development and provided molds for the subsequent electroplating (Arauna, $25~\hbox {g L}^{-1}$ Au) step, which was timed to reach a metal thickness of approximately $3~{\upmu }\hbox {m}$. The SU-8 resist mold was removed by means of a highly reactive plasma etching step (R3T STP 2020, Muegge GmbH, Germany), followed by cleaning the substrate in isopropanol, acetone, and deionised water. Wet-chemical etching of the gold seed layer in potassium iodide (${\mathrm {KI}}/{\mathrm {I_2}}$) solution and chromium in a permanganate based etch solution (Cr-etch-200, MicroChemicals GmbH) revealed a clean glass surface in unplated areas. For the wet-chemical etching steps, the bottom side of the substrate was protected by unexposed, soft baked $4~{\upmu }\hbox {m}$ thick resist (AZ4533, MicroChemicals GmbH), which was subsequently stripped. The average roughness of the gold metal structures was $R_{\mathrm {q}}\approx 0.13~{\upmu }\hbox {m}$, as determined by white light interferometry measurements. Three sheets (nominal thickness per sheet: $55~{\upmu }\hbox {m}$) of permanent dry film resist (Ordyl SY355, Elga Europe), yielding a total thickness of $165~{\upmu }\hbox {m}$ were laminated to the top face of the substrate, using an office hot roll laminator (Photonex-Sync 235/325, GMP Co. Ltd., Korea) at a temperature of 85$^{\circ }$C^52^. The dry film resist served two purposes. Firstly, the fluidic channels and reservoirs were formed from it by means of UV lithography. Secondly it provided an adhesive bond interface for the top glass substrate. As reported in^52–54^, the optimum dosage ($180~\hbox {mJ cm}^{-1}$ to $280~\hbox {mJ cm}^{-1}$) for the UV exposure of the dry film resist, and bond temperature of (80$^\circ$C to 120$^{\circ }$C), were critical for the subsequent full wafer adhesive bond. However, as reported by Mueller et al.^55^ and in agreement with our experience, optimal post-exposure parameters were critical as well. A high bond yield was only achieved with dry film resist not fully cross-linked, thus still plastic and compliant enough to facilitate a firm bond with the glass substrate when subjected to heat ($>T_{\mathrm {g}}$) and pressure. A 0.2 mm thick cover glass substrate (D 263 T eco, Schott) was bonded to the structured dry film resist by applying constant force and temperature to seal the chip, using a hot embossing tool (EVG510 HE, EV Group, Austria). The ODNP probe head assembly is pictured in Fig. 6c.

## Supplementary Information

Supplementary Information.
