# Supplementary material for: Microfluidic Overhauser DNP chip for signal-enhanced compact NMR
Source: Sci Rep. 2021 Feb 25;11:4671. doi: 10.1038/s41598-021-83625-y (PMC7907115; doi:10.1038/s41598-021-83625-y)
Supplement: Supplementary file 1 — Supplementary Information. [file 41598_2021_83625_MOESM1_ESM.pdf]

Supplementary materials: Microfluidic Overhauser DNP chip for signal-enhanced compact NMR

Sebastian Z. Kiss, Neil MacKinnon, Jan G. Korvink*
Institute of Microstructure Technology, Karlsruhe Institute of Technology, Hermann-von-Helmholtz-Platz 1, 76344, Eggenstein-Leopoldshafen, Germany
*jan.korvink@kit.edu

1 Microwave resonator design considerations and characterisation

The unloaded resonance frequency f_0 of a microstripline relates to its length as

$$L = m\frac{\lambda}{2} = m\frac{c}{2f_0\sqrt{\varepsilon_l}}, \tag{1}$$

for length L, wavelength λ, speed of light c, and integer mode number $m \in \{1, 2, \cdots\}$. The unloaded quality factor Q_0 for a microstripline resonator can be determined from

$$Q_0 = \frac{\beta}{2\alpha} = \frac{\beta}{2(\alpha_\mathrm{c} + \alpha_d)}, \tag{2}$$

with propagation constant $\beta = 2\pi/\lambda$, and total attenuation constant α accounting for the predominant loss mechanisms involved, here the dielectric loss α_d and conductor loss α_c. The attenuation due to conductor loss can be estimated by

$$\alpha_\mathrm{c} = \frac{R_\mathrm{s}}{Z_0 w}, \tag{3}$$

and surface resistivity

$$R_\mathrm{s} = \sqrt{\frac{2\pi f_0 \mu_0}{2\sigma_\mathrm{Au}}}, \tag{4}$$

with $\mu_0 = 4\pi \times 10^{-7}\,\mathrm{H\,m^{-1}}$ the permeability of free space. For a microstrip resonator made from gold with an electric conductivity σ_Au and an operating frequency of $f_0 = 14\,\mathrm{GHz}$, the attenuation due to conductor loss is determined to be $\alpha_\mathrm{c} \approx 1.7\,\mathrm{Np\,m^{-1}}$. The attenuation due to dielectric loss can be estimated by

$$\alpha_\mathrm{d} = \frac{k_0 \varepsilon_\mathrm{r}(\varepsilon_\ell - 1)\tan\delta}{2\sqrt{\varepsilon_\ell}(\varepsilon_\mathrm{r} - 1)}, \tag{5}$$

which results, for the substrate properties given in Table S2, in $\alpha_\mathrm{d} \approx 2.44\,\mathrm{Np\,m^{-1}}$. As determined by Equation (2), the unloaded qualty factor is expected to be $Q_0 \approx 75$. The feed line capacitively excites the $\lambda/2$-resonator via a narrow air gap of nominal width $C_\mathrm{gap} = 25\,\mu\mathrm{m}$. The gap is folded and forms a distributed interdigital capacitance C_κ. By adjusting the geometry of the coupling gap, the value of C_κ and therefore key parameters of the resonator can be tailored. From transmission line theory[1] the value for C_κ, required for critical coupling ($\kappa \approx 1$), can be determined as follows

$$C_\kappa = \sqrt{\frac{\pi\kappa}{2Q_0}}\frac{1}{2\pi f_0 Z_0}. \tag{6}$$

For the above mentioned operating conditions and $Q_0 \approx 75$, the desired coupling capacitance is found to be $C_\kappa \approx 0.03\,\mathrm{pF}$. As apparent from Equation (6), C_κ has a strong impact on the resonator's coupling coefficient κ, resonance frequency, as well as the impedance matching. For example, for decreasing quality factors, the resonator needs to be coupled tighter, i.e., increasingly higher values of C_κ are necessary in order to meet the condition of $\kappa \approx 1$ (assuming f_0 and Z_0 remaining constant). However, the practically more relevant effect is a significant change of the resonator's unloaded resonance frequency, as determined via Equation (1), once the resonator is capacitively coupled to the feed line. The required capacitance of $C_\kappa \approx 0.03\,\mathrm{pF}$ can be translated into values for C_gap, C_w, C_l for a corresponding gap geometry by the following equations[1]

$$C'_\kappa = C_0 + \frac{\varepsilon_\ell 10^{-3}}{18\pi}\frac{K(\hbar)}{K'(\hbar)}(N-1)C_\mathrm{l} \tag{7}$$

$$\frac{K(\hbar)}{K'(\hbar)} = \frac{1}{\pi}\ln\left[2\frac{1+\sqrt{\hbar}}{1-\sqrt{\hbar}}\right] \tag{8}$$

$$\hbar = \tan^2\left[\frac{\pi C_\mathrm{w}}{4(C_\mathrm{w} + C_\mathrm{gap})}\right], \tag{9}$$

in which C_0 denotes the coupling capacitance of a plain, non-interdigitated, end-coupled resonator. For an interdigital coupling capacitor composed of $N = 3$ fingers with an approximated finger width of $C_w = 137\,\mu m$ and a minimal coupling gap width of $C_{gap} = 25\,\mu m$, the required finger length can be estimated by Equations (7) to (9) to be $C_l \approx 80\,\mu m$. Table S2 summarises the estimates of the most important design variables for the resonator, in particular the gap geometry.

To investigate the EM field properties of the resonators, driven numerical EM simulations were performed. The simulation model included the microstrip feed line and the coupling gap structure on a 0.3 mm thick glass substrate, as well as the MW resonator made from 3 µm thick gold. For accurate results, the SMA connector and the MW fixture had to be included into the model, as well as loss effects for all involved materials ($\tan\delta_{glass} = 0.01$, $\tan\delta_{teflon} = 0.001$). While sweeping model parameters, the distance between the centre of the resonator and the signal launch point at the substrate's edge was kept constant at 35 mm. For continuous MW excitation, a 50 Ω wave port, defined on the front facet of the coaxial connector, was used. The whole structure was placed inside a box-shaped domain to model the surrounding air, whereas absorbing boundary conditions suppressed any back scattering. A free tetrahedral mesh was employed and iteratively refined to resolve the structure accurately, e.g., for small geometrical features such as the interdigitated finger of the distributed coupling capacitor. Figure S2 compares the EM field distributions, at resonance, of the two resonator designs. Shown are the electric field magnitude as well as the H_x-field, i.e, the x-component of the MW magnetic field, being orthogonal to the DC B_0-field of the permanent magnet.

2 Design parameters

For the NMR resonator, the material and geometrical design parameters employed are listed in Table S1, and for the EPR resonator in Table S2.

Table S1. Design variables and values as used for a typical resonator.

parameter	symbol	typical value
relative permittivity	ε_r	6.3 at $f = 5$ GHz
effective permittivity	ε_ℓ	4.52
dielectric loss tangent	$\tan\delta$	0.01 at $f = 5$ GHz
substrate height	h	300 µm
coupling gap width	C_{gap}	25 µm
finger length	C_l	100 µm to 350 µm
finger width	C_w	137 µm
width microstrip	w	411 µm
width constriction	w_c	100 µm
length resonator	L	3.45 mm to 4.60 mm
length constriction	L_c	1 mm and 1.5 mm
electr. conductivity gold	σ_{Au}	$4.098 \times 10^7\,S\,m^{-1}$

Table S2. Estimated values for the design variables of a type 1 $\lambda/2$-resonator featuring a three finger interdigital coupling capacitor. The following assumptions were made: critical coupling $\kappa = 1$, characteristic impedance of $Z_0 = 50\,\Omega$, a resonance frequency of $f_0 = 14$ GHz, a transmission line width of $w = 411\,\mu m$.

ε_ℓ (a. u.)	L (mm)	Q_0 (a. u.)	C_κ (pF)	C_{gap} (µm)	C_w (µm)	C_l (µm)
4.52	5	75	0.03	25	137	80

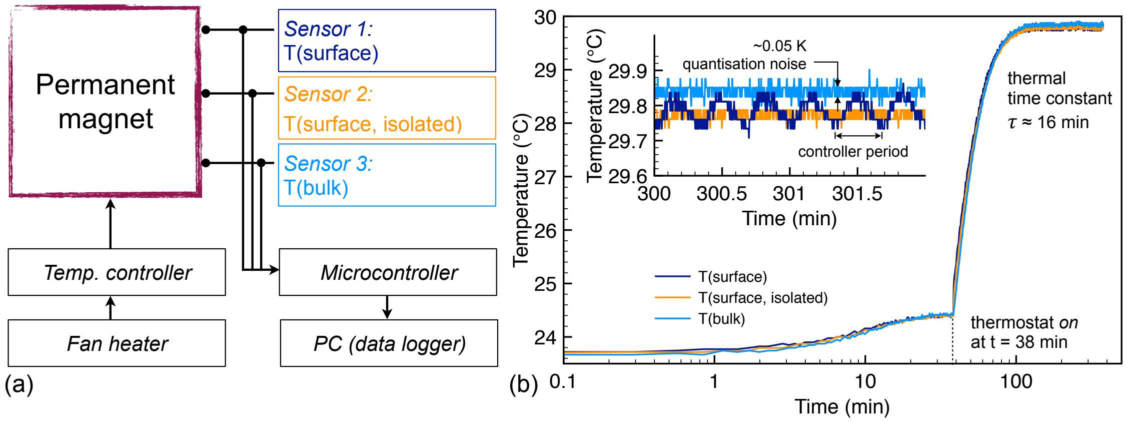

Figure S1. (**a**) Block diagram of the thermal stabilisation control of the permanent magnet. (**b**) Measured temperature data over a period of 370 min. Figure used with permission[2].

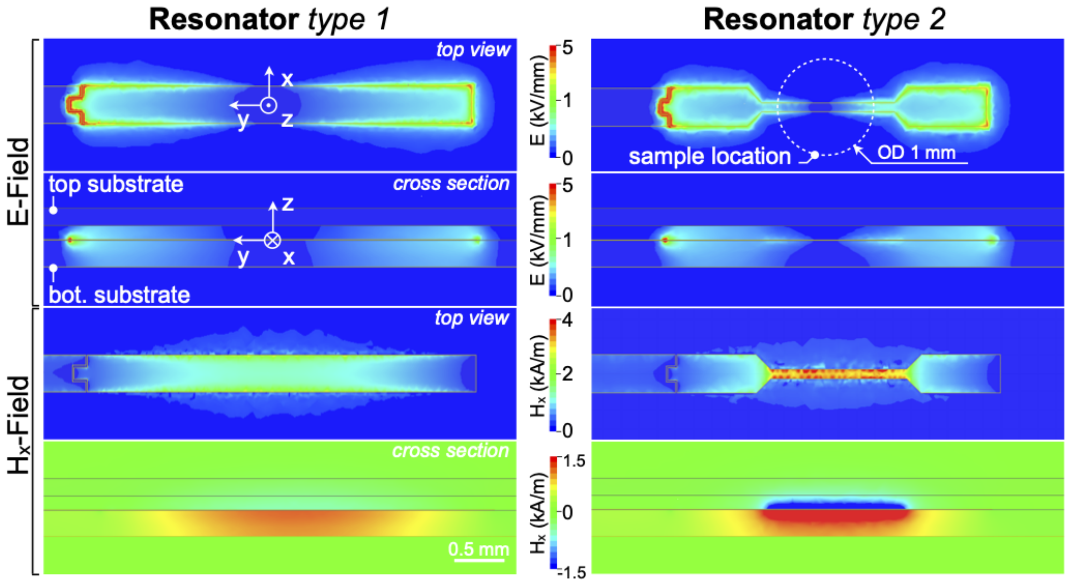

Figure S2. Simulated EM field distributions for resonator types 1 and 2. Shown are the electric field magnitude E, as well as density plots for the x-component of the magnetic field H at resonance ($f_0^{\text{type1}} = 14.14\,\text{GHz}$, $f_0^{\text{type2}} = 14.36\,\text{GHz}$). Input power was set to 1 W. Figure used with permission[2].

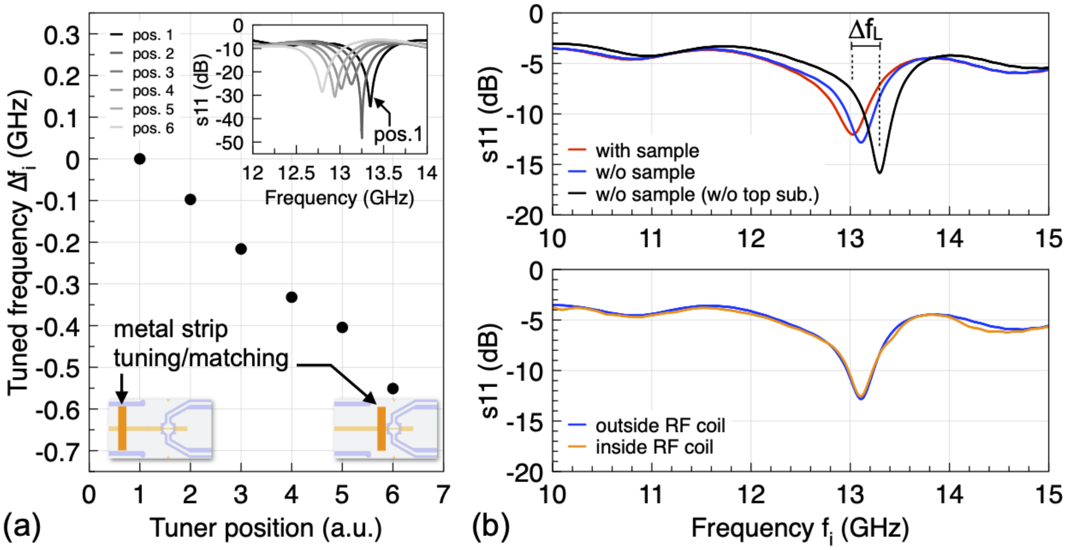

Figure S3. (**a**) Resonance frequencies Δf_i measured from a single resonator for different positions i of the metal tuning/matching strip. Tuner positions are numbered to semi-quantitatively indicate their position, with 1 being far away from the coupling gap region (no tuning/matching effect) and 6 being the point of max. insertion. (**b**, top) Representative measured shift in frequency $\Delta f_L \approx -300\,\text{MHz}$ due to sample loading (water), the presence of the dry film resist and the top substrate. (**b**, bottom) Spectra of the reflection coefficient obtained from the same MW resonator measured inside and outside the RF coil. Figures used with permission[2].

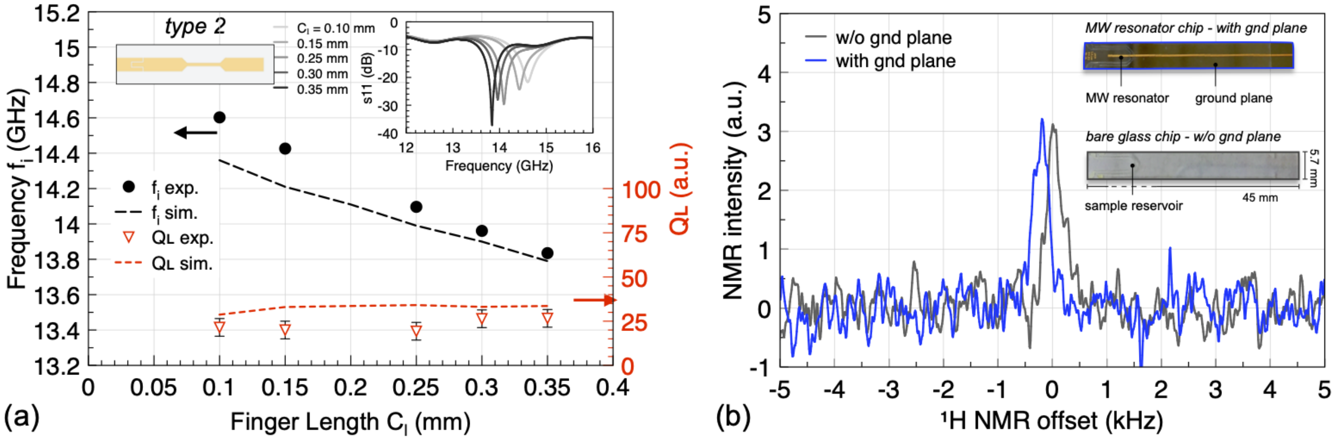

Figure S4. (**a**) Measured (points) and simulated (dashed lines) resonance frequencies f_i and loaded quality factors Q_L as a function of the finger length C_l; each data point represents a separate resonator. The inset shows the corresponding S_{11} spectra of the resonators. The presented S_{11} data were measured from resonators with empty sample reservoirs. Nominal geometry values are: $C_{gap} = 25\,\mu m$, $w_c = 100\,\mu m$, $L_c = 1.5\,mm$, $L = 3.555\,mm$. (**b**) ^{1}H-spectra measured from two different glass inserts (with and without metallisation), show no significant difference in NMR intensity and line shape. Insets show photos of the bare glass chip and the metallised version. The spectra were acquired from 130 nL of water with the electrical shim-system turned off, i.e., for the measurement the electrical shim system was not engaged. Figures used with permission[2].

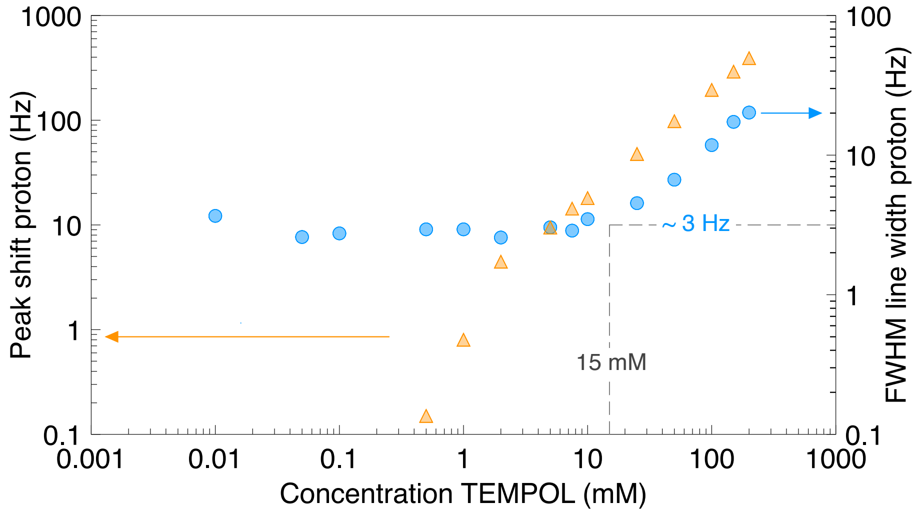

Figure S5. Measured ^{1}H NMR peak shifts and FWHM line widths as a function of TEMPOL concentration. Measurements were performed on a 500 MHz NMR spectrometer (Avance III, Bruker, Germany) with the radical dissolved in DI water and Acetone (50/50 deuterated) employed for reference.

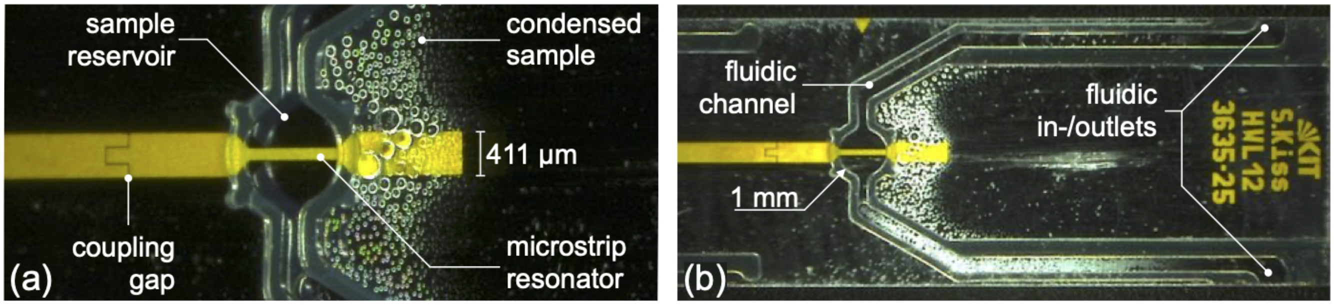

Figure S6. (a), (b) Photographs of a MW resonator chip after operation at MW input power levels above 1 W. In this particular case the adhesive bond interface (dry film resist/glass surface) failed due to thermal/mechanical stress. As a result, the sample solution condensed outside the actual reservoir region, whereas parts of the aqueous sample evaporated via the unsealed inlets and outlets of the chip. Figure used with permission[2].

3 Experimental setup and procedure

The following additional components are permanently used for the characterisation experiments: Isolator (PE8305, Pasternack, USA), the circulator (K36-1FFF, Aerotek, Thailand), with a 50 Ω termination at port 3, a wide band (5.9 GHz to 18 GHz) MW amplifier (ZVE-3W-183+, 33.5 dB gain at 14 GHz, Mini-Circuits, USA), the shim current driver, as well as a lock-in amplifier. For MW irradiation a solid-state MW sweep oscillator (HP8350B, RF plug-in 0.01 GHz to 26.5 GHz) is employed. To ensure correct MW power level-settings, the nominal output power range (−5 dBm to 20 dBm) of the MW generator, with and without the MW amplifier, was referenced by a MW power meter (ML2495A + MA2442D sensor, Anritsu, Japan). In combination with a set of MW attenuators (6 dB Macom 2082-6041-6, 10 dB Suhner 661019AA, 20 dB Narda microPad 4749-20) output powers within a range of 0.2 mW to 3.45 W can be achieved. A one-port reflectometer setup (see gray lines in the manuscript Figure 5 (c)) allows to determine the MW resonator's frequency by using a digital oscilloscope in xy-mode. The start and stop timings for switching/gating the MW irradiation on/off are controlled by the NMR spectrometer console (Avance III, Bruker, Germany), by connecting its TTL trigger output signal to the "pulse-in" input port of the MW source. Typical MW pulse lengths for steady state ODNP experiments were set to values between 300 ms to 1000 ms, with rise/fall switching times as specified for the MW source of 15 ns. The NMR RF coil is impedance matched and frequency tuned, by a remote capacitive tuning/matching PCB, using fixed as well as variable trimmer capacitors. Impedance matching is achieved by symmetric, serial capacitors of approximately 82 pF on each side. To achieve resonance at around 20 MHz, a total capacitance, parallel to the RF coil, of approximately 320 pF is necessary.

The setup is EPR-ready and contains an optional reference arm, including a MW phase-shifter, a variable attenuator, a zero-bias Schottky detector diode (R451533000, Radiall), as well as an EPR modulation coil. All parts of the MW bridge are connected by semi-rigid, handformable 50 Ω MW coaxial cable (Sucoform 141, Huber+Suhner, Switzerland).

ODNP measurement procedure

In the following the procedure and prearrangements for a typical ODNP experiment are described.

1. For reproducible experiments, particularly for very long measurements, the permanent magnet should be preheated above ambient temperature as described in the *Permanent magnet* Section of the manuscript (time duration until thermal equilibrium is approximately 70 min). This ensures similar B_0-field values before each measurement and reduces temperature induced drift over time.

2. As the thermal NMR signal intensities are very low – due to the low field strength of the magnet and low sample volumes (typically 130 nL) – it is very important to shield out external noise from being picked up by the RF coil, in order to prevent the low NMR signals from being obscured by the presence of an excessive noise floor in the recorded spectra. Therefore, the magnet as well as the environmental box (encased by copper) including all exposed RF feed structures, such as the tuning and matching circuit, must be shielded and properly connected (no ground loops) to the ground potential of the NMR RF preamplifier.

3. In order to check the NMR detection of the system, an NMR test measurement can be performed. The *xy*-linear stages are employed to accurately position the sample at the sweet spot of the magnet. The NMR pilot measurements are ideally performed on microfludic chips featuring a large sample reservoir providing sufficient spin concentration.

4. The permanent magnet's field coils need to be connected to the amplified auxiliary port of the lock-in amplifier, to provide the DC *B*-field sweep, as known from CW EPR experiments. For phase sensitive EPR detection the modulation coils are connected to one of the signal output ports of the lock-in amplifier. For details, see the main manuscript, Section *ODNP setup and signal processing*.

5. The MW source is timed and triggered by the NMR console. Therefore, it is necessary to connect the trigger lines from the NMR console to the MW source's blank input port and to include the corresponding trigger command line inside the NMR pulse program (see the main manuscript, Section *ODNP setup and signal processing*, for details).

6. A MW resonator chip with known unloaded resonance frequency f_0 is selected and loaded with sample solution. Due to the change in the dielectric environment of the resonator upon sample loading, the resonator's resonance frequency shifts (the change in frequency Δf_L depends on the sample properties and the type of fluidic chip (e.g., relative permittivity, radical concentration, diameter and shape of the sample reservoir); typical Δf_L for aqueous solution is about −300 MHz) and needs to be re-measured (temporarily unplug the trigger lines from the "pulse-in" port at the MW source, see reflectometer setup (gray lines) in Section *Material and Methods*). For a centered quasi EPR spectrum (MR intensity vs. ΔB_0) the resonance frequency of the chip is ideally fine-tuned (see tuning/matching strip, Fig S3) as close as possible to the frequency-equivalent of the magnet's static B_0-field value (typically around 13.84 GHz).

7. Carefully, insert the MW resonator chip into the RF coil and attach the MW fixture via two screws to the mount module. Connect the semi-ridged MW coaxial cable to the MW SMA input of the resonator and position the probe head at the sweet spot of the magnet using the linear stages.

8. The equivalent ^{1}H NMR Larmor frequency is calculated from the set MW resonance frequency f_{L}, in order to set the NMR transmitter frequency at the NMR console by typing the command `SFO1` followed by the frequency value.

9. Enter the "wobble-mode" in TopSpin™ by issuing the command `wobb` to tune and match the NMR coil at set f_{n}, by manually trimming the variable capacitors of the RF coil's tuning and matching board.

10. Set up and check the connections of the MW bridge as shown in the main manuscript (Section *ODNP setup and signal processing*). Set the MW output power level at the source to 20 dBm. Switch on the MW signal output of the MW source by pushing the "RF button" (note that the MW sweeper is not yet transmitting a signal, as the blank input port of the device is active, see the main manuscript, Section *ODNP setup and signal processing* for details).

11. Setting up the NMR acquisition parameters: In TopSpin™ the command `edpa` is entered to set the pulse acquisition parameters. The spectral width parameter of initial experiments is set relatively large, in order to cover a sufficiently broad spectral region. A single $\pi/2$-pulse experiment is issued by typing the command `zg`, followed by the command sequence `epf, apk, abs, dpl1` for post-processing of the spectrum. Set time delays and duration for DNP.

12. The command `gs` is issued to enter the gradient shim module, in order to interactively observe each acquired spectrum of a single shot NMR $\pi/2$-pulse experiment. As the MW experiments are acquired and spectra are continuously updated, the electrical current and hence the magnetic field strength of the magnet's auxiliary coil is iteratively changed by small increments until the resonance condition for EPR, manifesting itself in a large (negative) hyperpolarised NMR signal peak. The electrical current is fine-tuned for a maximal NMR signal peak intensity.

13. Once an NMR signal becomes prominent above noise, the single-shot signal is observed via the interactive gradient shim window in TopSpin™ and shimmed using the implemented five channel shim coils. The electrical current through each shim coil is fine tuned via the precision potentiometers at the shim current driver for maximal NMR signal intensity.

14. For high-power ODNP experiments, the MW amplifier is added to the MW bridge (see the main manuscript, Section *ODNP setup and signal processing*), amplifying the MW signal from the source to a maximum power level of around 3.45 W at 13.8 GHz.

References

1. Bahl, I. J. *Lumped Elements for RF and Microwave Circuits* (Artech House, 2003).

2. Kiss, S. Z. *Overhauser DNP probes for compact magnetic resonance*. Ph.D. thesis, Albert Ludwig University Freiburg, Freiburg (2019).
